# Detection of heavy metals and VOCs in streambed sediment indicates anthropogenic impact on intermittent streams of the U.S. Virgin Islands

**DOI:** 10.1038/s41598-023-44455-2

**Published:** 2023-10-11

**Authors:** Brittany V. Lancellotti, David A. Hensley, Race Stryker

**Affiliations:** 1 Virgin Islands EPSCoR, University of the Virgin Islands, Kingshill, VI USA; 2 Agricultural Experiment Station, University of the Virgin Islands, Kingshill, VI USA

**Keywords:** Environmental impact, Environmental impact

## Abstract

Global surges in industrialization and human development have resulted in environmental contamination. Streambed sediment contamination threatens ecological and human health due to groundwater leaching and downstream contaminant mobilization. This is especially true in the wider Caribbean region, where streambed sediment contamination is understudied. In the current study, we assessed human impact on intermittent streams by measuring heavy metals and volatile organic compounds (VOCs) in streambed sediment on St. Croix in the United States Virgin Islands (USVI), where intermittent streams receive limited conservation and research attention. In contrast to our hypothesis that streambed sediment pollutant concentrations would be higher in developed, compared to undeveloped areas, contaminant concentrations did not vary significantly according to land cover. Elevated lead, mercury, and zinc concentrations were correlated with commercial building density, suggesting an unnatural origin of these elements in streambed sediment. At some sites, levels of arsenic, cadmium, chromium, nickel, lead, thallium, or zinc exceeded regulatory limits. The most prevalent VOCs at both developed and undeveloped sites were benzene and toluene. Sub-groups of heavy metals identified by principal component analysis indicated potential pollution sources, including fuel combustion (chromium, nickel, arsenic, selenium), vehicle exhaust, oil refining, and gasoline leaks (2-butanone and xylenes), and plastics (acetone and styrene). Our results suggest USVI intermittent streams require further research attention and intervention strategies for pollution reduction.

## Introduction

The presence of environmental contaminants (i.e., substances at concentrations associated with adverse effects on water, air, soil, or biotic communities) in natural systems can indicate anthropogenic impact. Soil and sediment are major pollutant sinks and are particularly vulnerable to contamination, especially as global industrialization and development persist^1^. Contamination of streambed sediment poses distinct threats to human and ecological health, as contaminants can leach into groundwater systems or adhere to fine sediments and become mobilized downstream. Streambed sediment contamination has been reported in many parts of the world, but this topic is generally understudied in most regions^2^. Signs of environmental stress, including negative ecological effects of toxic substances, have been documented in the wider Caribbean, one of the most tourism-dependent regions of the world^3–5^.

The United States Virgin Islands (USVI), an eastern Caribbean territory of the U.S., has undergone substantial land use shifts in recent decades due to increases in population and land development^6–8^. Intermittent streams, locally referred to as ‘guts’, are important conduits that connect terrestrial and nearshore marine environments. They can play an essential ecological role by providing critical habitat for migratory birds and freshwater invertebrates, fish, shrimp, and amphibians, some of which are endemic species^9,10^.

Steep terrain and thin soils characteristic of the USVI result in rapid surface runoff generation, low groundwater recharge, and thus, intermittent flow in most streams^11^. Intermittent streams host sporadic, flashy discharge events that may trigger substantial pollutant pulses. As intermittent streams of the USVI are not regulated under an actionable policy framework, they are vulnerable to contamination^12,13^. For example, USVI streams are susceptible to contamination from improper sewage and stormwater management, nutrients and sediment-rich runoff from impervious and unpaved roads, and illegal trash disposal resulting from inadequate solid waste management^13^. However, due to their ephemerality and non-navigability, USVI streams do not receive adequate research attention^14^. Published data on sediment pollution within the wider Caribbean region are generally limited^3^. Within the USVI, research assessing sediment pollution has almost exclusively sampled marine sediment. A few studies have been conducted within the USVI to evaluate the impacts of marine sediment, heavy metals, or other pollutants (e.g., butylins, polycyclic aromatic hydrocarbons, pesticides, polychlorinated biphenyls) on nearshore marine ecosystems^15–20^. Additionally, one 1986 study measured lead concentrations in urban road dust within the USVI^21^.

Soils are the main sink for heavy metal pollution^22^. While heavy metals naturally exist in soil from parent rock weathering, they can accumulate at toxic levels if rates of anthropogenic inputs exceed the natural weathering process. Various calculation methods are available to assess the degree of heavy metal enrichment in soils (e.g., Enrichment Factor, Geoaccumulation Index, Modified degree of contamination)^23^. Common sources of heavy metal soil toxicity are wastewater treatment plants^24^, heavy industry (e.g., oil and bauxite refining), vehicles, landfills, and agriculture^25^, all of which are present on St. Croix, USVI. Heavy metals are transported through stream systems as species dissolved in water or adsorbed to suspended sediment and can therefore be deposited onto the streambed. Most metals cannot be chemically or microbially degraded and can therefore persist in the soil environment long-term^22^. Through contact with, or ingestion of contaminated soil, human exposure to toxic levels of heavy metals can cause kidney, gastrointestinal, and immune system dysfunction, birth defects, and cancer^22^. Soil heavy metal toxicity can also negatively impact stream ecosystem health by decreasing litter decomposition^26^ and altering biogeochemical processes^27^, plant growth^28^, and benthic invertebrate community structure^29^.

According to the U.S. Environmental Protection Agency^30^, volatile organic compounds (VOCs) are a large group of organic chemicals that have high vapor pressure and are resistant to degradation. These chemicals are emitted to the atmosphere both naturally, by soil microorganisms, plants, and volcanic eruptions, and unnaturally, by a variety of anthropogenic processes. Human-produced VOCs are known to have toxic, mutagenic, and carcinogenic effects on human and ecological health^30^. Although VOCs are the most widespread pollutants and are highly mobile in the environment, human-produced VOCs in soils and sediments are understudied^31^. Volatile organic compounds are byproducts of the oil refining process and the production of pesticides, paints, glues, dyes, etc. A variety of products emit VOCs, including cleaning supplies, cosmetic products, and building materials. Additionally, VOCs are primary components of oil products and fuel combustion, and accidental oil spills are a source of VOC contamination^30^. According to the U.S. Geological Survey^32^, VOCs exist in various phases in the environment (i.e., gas, aqueous solution, sorbed to soil, nonaqueous-phase liquid). Although VOCs have low water solubility because of their tendency to quickly volatilize, if they volatilize during transportation to the saturated zone, VOCs can become dissolved in water and contaminate groundwater. Highly soluble VOCs can travel long distances as aqueous solutions, whereas hydrophobic VOCs may adsorb to organic matter and thus remain at the source. Soil microorganisms degrade certain VOCs, but VOCs generally persist in groundwater for extended periods^32^. Select VOCs adversely affect the human liver, kidneys, spleen, stomach, and several human organ systems (e.g., nervous, circulatory, reproductive, immune, etc.). Additionally, some VOCs have carcinogenic effects on humans and animals. As such, their unnatural presence in the environment is of critical concern^33,34^.

Human impact on intermittent streams within the USVI remains poorly described^12,35^, thus representing a critical knowledge gap. Especially as development continues to increase rapidly throughout the territory, an improved understanding of human land use and concentrations of heavy metals and organic contaminants in streambed sediment is essential. Previous research efforts have documented strong, positive correlations between human land use and streambed sediment heavy metal and organic contaminants, suggesting the presence of these pollutants could serve as a signal of human impact on intermittent stream systems^2,36–39^. We therefore assessed chemical pollution impacts on intermittent streams on St. Croix, USVI. We hypothesized that streambed sediment pollutant levels would exhibit a spatial pattern consistent with higher concentrations in developed, compared to undeveloped areas. Alternatively, we hypothesized that contamination would be widespread and would not follow a strict pattern according to land cover. To test our hypotheses, we measured heavy metals and VOCs in dry streambed sediment across gradients of land cover, soil type, and microclimate on St. Croix, USVI, thereby providing the first dataset of its kind for the USVI.

## Materials and methods

### Study area

The USVI, located 65 km east of Puerto Rico and within the northern portion of the Lesser Antilles, consists of St. Croix, St. Thomas, St. John, Water Island, and several small, uninhabited islands. The present study took place on the island of St. Croix, which is the largest (218 km^2^) and the second most densely populated (98 persons/km^2^) of the USVI^40^. The climate of St. Croix is subtropical, with a mean annual temperature of 26.4 °C^41^. The Köppen climate class for the island is “tropical savanna, dry winters”^42^. Mean annual rainfall on St. Croix exhibits a strong longitudinal gradient, with higher mean annual rainfall on the west end of the island (147.5 cm), compared to the east end (94.3 cm)^43^. Rainfall typically occurs in short duration showers with low accumulation rates^44,45^. More than half of the annual cumulative rainfall occurs during August-November^43^. Tourism rapidly expanded in the 1950s and is currently the territory’s leading economic sector^46^. St. Croix has a history of plantation agriculture (ceased in the 1960s) and heavy industry, including a crude oil refinery (operational 1966–2012 and 2019–2021), a bauxite production plant (operations ceased in 2002), an ethanol dehydration plant (2007-present), and a rum distillery (2011-present). Industrial activities are mostly concentrated within an industrial park on the south shore.

### Site selection using a geographic information system

To identify potential study sites, we conducted an island-wide geospatial survey to locate intermittent streams using Quantum Geographic Information System (QGIS) software^47^. We identified potential study sites by delineating streams and catchments using the SAGA channel network and drainage basins tool with a Strahler order of six^48^. We then conducted site visits to verify accessibility via roads or pathways. Site selection was finalized by choosing 30 locations that spanned an east–west, north–south, and elevation gradient, to capture a wide range of microclimates, soil types, and land cover. The exact location of each finalized sampling site was recorded with a portable Global Positioning System (GPS) device.

**Figure 1:**
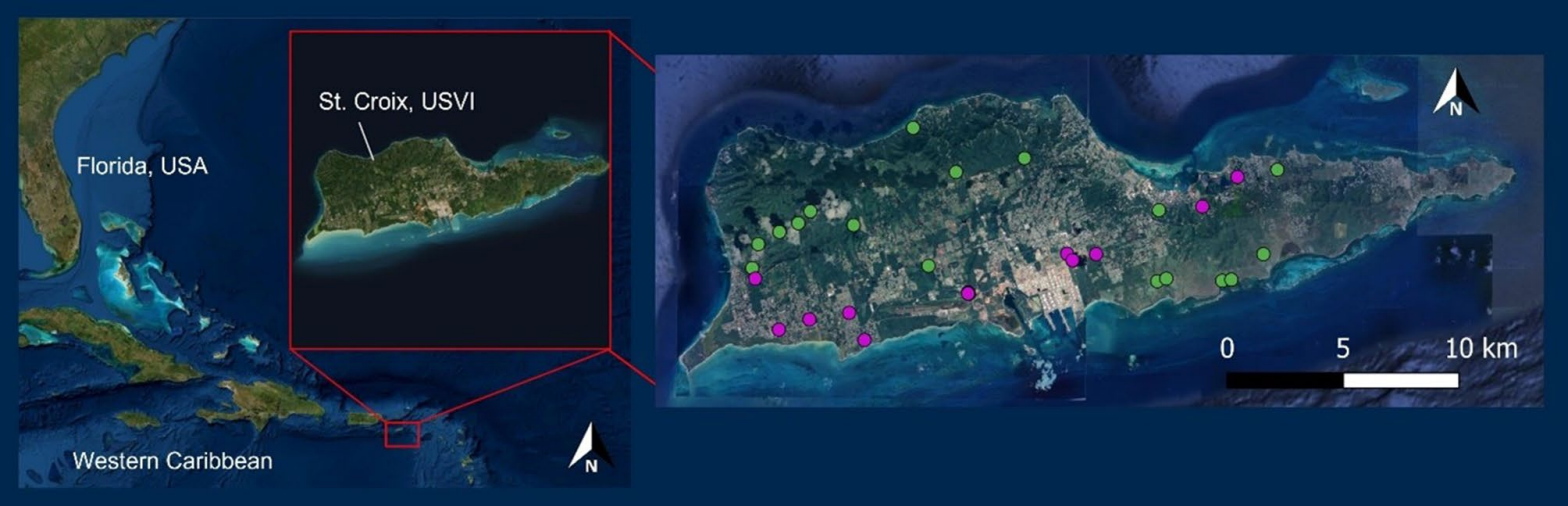
Google satellite imagery of the study area, St. Croix, United States Virgin Islands, with developed and undeveloped sampling sites shown in magenta and green, respectively. Map created using QGIS software, version 3.28.11, https://www.qgis.org/en/site/forusers/download.html.

We used remotely sensed land cover classification data from 2018^49^ and grouped similar land cover classes together to categorize potential sites as developed or undeveloped. The developed land cover class included a range of development intensities (low–high) and developed open space. The undeveloped classification included wetland, forest, shrub, and rangeland. Our finalized selection included 13 developed sites and 17 undeveloped sites (Fig. 1). To determine site-specific soil characteristics, we used geospatial soil taxonomy data from the National Resources Conservation Service (NRCS) online Web Soil Survey^50^. To separately calculate the commercial and residential building density within each study watershed, we utilized publicly available satellite imagery that included building footprints (i.e., building outlines drawn along exterior walls) from the Federal Emergency Management Agency^51^. We manually selected for buildings with residential or commercial use and categorized them accordingly. Industrial buildings were included in the commercial use category. We then calculated the residential and commercial building density of each study watershed by dividing the number of buildings in each category by the watershed area.

### Site descriptions

We calculated geospatial properties to provide a general characterization of the study sites (Table 1). For each study site, we calculated the distance to the nearest roadway (distance to road), as vehicle pollution can contribute significantly to sediment contamination^52^. As contaminant yields can vary with catchment size^53^, we calculated the distance from each study site to the closest shoreline (distance to shore) to represent upstream catchment area. Soils at the study sites were classified as Mollisols or Inceptisols, belonging to the Annaberg-Cramer complex (*isohyperthermic Typic Haplustolls*), Arawak series (*isohyperthermic Typic Calciustolls*), Carib series (*isohyperthermic Mollic Endoaquepts*), Glynn series (*isohyperthermic Typic Argiustolls*), and Hesselberg series (*isohyperthermic Petrocalcic Paleustolls*)^50^.

**Table 1:** Summary statistics of spatial characteristics grouped by land cover.

Land cover	Statistic	Elevation	Distance to road	Watershed area	Distance to shore
		m	m	km^2^	km
Developed	Mean	**12**	16.2	11,532.9	1.2
Undeveloped		**30.5**	26.8	6793.5	1.4
Developed	Range	3.1–26.9	3.6–47.3	1569.7–26,163.1	0.29–2.3
Undeveloped		4.6–98.4	1.9–219.2	557.71–26,163.1	0.14–3.3
Developed	CV	78.6	68.7	89.4	49.3
Undeveloped		104.8	190.3	95.6	78

Bold indicates a significant difference between land covers (t-test; P < 0.05).

### Sample collection

We collected sediment from 30 dry streambeds on 14 April 2022 (16 samples) and 2 June 2022 (remaining 14 samples). Weather conditions remained steady (very limited precipitation) between sampling campaigns. At each site, we collected 10 sediment samples (0–5 cm) at random, with a metal-free plastic spade, within an area of approximately 10 m^2^. To prevent contamination, the metal-free spade was cleaned with a fresh microfiber wipe between sites. Within a site, the 10 sediment samples were composited inside a 3.8 L plastic bag and homogenized by manual mixing (i.e., shaking, massaging, kneading by hand for approximately one minute). In transit to the laboratory, the composite samples were stored on ice in sealed 1 L plastic bags. The composite samples were then sub-sampled (150 g; rocks and plant material removed) and shipped on ice in sealed plastic bags to the laboratory within 72 h of sampling.

### Recovery of analytes from streambed sediment

The streambed sediment samples were analyzed for 42 volatile organic compounds (VOCs) (Supplemental Table s-1), total petroleum hydrocarbons-gasoline range organics (TPH-GRO), 12 total heavy metals, total organic carbon (TOC), and soil moisture by an independent laboratory (Pace Analytical Testing Laboratories, St. Rose, LA, USA). Analysis of VOCs was completed with gas chromatography/mass spectrometry (GC/MS) using United States Environmental Protection Agency (USEPA) preparation methods 5035 and 5030b and analytical method 8260^54^ (low level detection). Analysis of TPH-GRO was completed using gas chromatography with flame ionization detection (GC-FID) with USEPA preparation methods 5035A and 5030B^54^ and analytical methods 8015^54^ and 8021^55^. Analysis of total (i.e., all elements that could become “environmentally available”) aluminum (Al), antimony (Sb), arsenic (As), cadmium (Cd), copper (Cu), lead (Pb), nickel (Ni), selenium (Se), silver (Ag), thallium (Tl), and zinc (Zn) was completed with inductively coupled plasma-atomic emission spectrometry (ICP-AES) using USEPA preparation method 3050 (a strong acid digestion technique) and analytical method 6010^54^. Total mercury (Hg) was measured using cold-vapor atomic absorption with USEPA method 7471^54^. TOC was determined with combustion catalytic oxidation using USEPA method 9060^56^.

### Sample analysis and quality control

Results were reported using a correction factor for soil moisture content. Percent soil moisture was determined using a gravimetric technique^57^. All results were reported in dry weight. The reporting limit was 0.016–72.7 mg kg^−1^ for metals, 5.3–11.6 ug kg^−1^ for VOCs, 2540–3020 ug kg^−1^ for TPH-GRO, 250 mg kg^−1^ for TOC, and 0.5% for soil moisture. Several quality control parameters were performed for quality assurance. For VOC and TPH-GRO analysis, each sample was spiked with a surrogate compound to evaluate extraction efficiency. All samples subjected to VOC analysis, plus a method blank, were spiked with Toluene-d8, 4-Bromofluorobenzene, and Dibromofluoromethane, and the recovery of all samples and the method blank ranged from 96 to 111%. All samples subjected to TPH-GRO analysis, plus a method blank, were spiked with 4-Bromofluorobenzene, and the recovery of all samples and the method blank ranged from 93 to 95%.

For each batch extraction, each analyte was subjected to a method blank analysis, and the results showed undetectable levels of the analytes in all method blanks. Each batch extraction for each analyte was also subjected to verification using a laboratory control sample (LCS) to check for interferences within the sample matrices. The LCS recovery was 86–100% for metals, 105–115% for VOCs that were detected in our sediment samples, 98% for TPH-GRO, and 92–96% for TOC. To evaluate the accuracy and precision of each analytical method, matrix spikes (MS) and matrix spike duplicates (MSD) were performed for each batch extraction of each analyte. The relative percent difference (RPD) between the MS and MSD for all metals ranged from 9 to 30. The matrix spike RPD for Al, Cd, Cr, Pb, Ni, and Se exceeded the control limit of 20, likely owing to the heterogeneous nature of the sediment samples. Of the VOCs that were detected in sediment samples, the matrix spike RPDs ranged from 55 to 83, and thus exceeded the control limit. The matrix spike RPD for TPH-GRO and soil moisture was 1 and 0, respectively. The matrix spike RPD for TOC was 40, thus exceeding the limit of 25 (likely due to sediment heterogeneity).

### Statistical analysis

Statistical analyses were performed in RStudio^58^. An alpha level of 0.05 was used to determine significance. Independent t-tests were performed to compare group means of measured parameters between land cover classifications. We used simple linear regression to investigate relationships between contaminants (i.e., heavy metals, VOCs, TPH-GRO; dependent variables) and soil properties (i.e., TOC and soil moisture; independent variables). We also performed simple linear regression to assess relationships between heavy metals and Al concentrations, as strong correlations between heavy metals and Al are argued to indicate lithogenic origin^20^. We jointly analyzed the dataset (N = 30) for all linear regression analyses.

For each site, we calculated the geoaccumulation index (Igeo) of each metal^59^. The Igeo is a widely used index that assesses the degree of heavy metal contamination within the context of background (i.e., naturally occurring) heavy metal levels. Igeo is calculated as follows:

$${\text{I}}_{{{\text{geo}}}} = {\text{ log}}_{{2}} \left( {{\text{C}}n/{1}.{\text{5 B}}n} \right),$$

where C*n* is the measured concentration of the metal, B*n* is the background concentration of the metal, and 1.5 is an index used to correct for differences in geologic properties between study sites^2^. We used mean crustal concentrations of heavy metals as values for background levels (B*n*)^60^, as reliably accurate background concentrations were not available for terrestrial environments on St. Croix, USVI^61^. The Igeo includes seven contamination classes: unpolluted (Igeo ≤ 0); unpolluted to moderately polluted (0 < Igeo ≤ 1); moderately polluted (1 < Igeo ≤ 2); moderately to strongly polluted (2 < Igeo ≤ 3); strongly polluted (3 < Igeo ≤ 4); strongly to extremely polluted (4 < Igeo ≤ 5); extremely polluted (5 < Igeo).

We performed principal component analysis (PCA), a dimensionality reduction tool, on correlation matrices (scaled to unit variance) of different groupings of the measured parameters using the *prcomp* function in the ‘stats’ package in RStudio. We used the dimensionality reduction feature of the PCA to i) identify suites of contaminants that contributed significantly to the variation in the dataset and ii) detect strong associations between contaminants (i.e., their co-occurrence in the environment), and thus, potential pollution sources^62,63^. We chose two final PCAs (one including only metal concentrations; one including only VOCs and TPH-GRO) that maximized the variance explained by the principal components (PCs), had eigenvalues > 1, and provided useful information about the data variability.

We used the PC loadings (i.e., coefficients of the linear combination of original values from which the PCs were constructed) from each PCA to identify the main drivers of each PC and strong associations between contaminants. The main drivers of each PC were identified as the variables that cumulatively accounted for at least 50% of variability within the PC (i.e., the variables with the largest loadings). We then grouped the contaminants accordingly. To test for underlying patterns in contaminant concentrations, we first plotted the PCA scores (i.e., original data multiplied by the rotation matrix; hereafter referred to as PC scores) of the first two PCs. Next, by color-coding, we clustered the observations according to various parameters that were expected to account for their spatial variability (e.g., land cover, soil order, TOC, elevation, distance to road, distance to shore, etc.). For continuous variables, we used Jenks Natural Breaks Optimization^64^ to group values into three main categories: “low”, “moderate”, and “high”.

We also utilized the dimensionality reduction feature of PCA for pattern recognition. For each of the PCs with eigenvalues > 1, we grouped PC scores greater than zero (i.e., sites that could be described as having features represented by that specific PC) into “lower”, “moderate”, and “elevated” categories using Jenks Natural Breaks Optimization. A highly negative loading indicates a lack of the variable(s) associated with that loading. Therefore, in the case of highly negative loadings, we grouped the positive PC scores associated with the highly negative loadings (sites lacking particular contaminants) according to Jenks Natural Breaks. For each PC, we mapped all PC scores in QGIS, including categorized positive and negative PC scores, and overlaid them on various geospatial data, including heatmaps of commercial and residential building density^65^. We performed Pearson correlations to assess relationships between positive PC scores and the commercial and residential building density of the watershed associated with each sample.

## Results

### Streambed sediment heavy metal concentrations and soil properties

Al, Cr, Cu, Pb, and Zn were detected in all 30 streambed sediment samples. Ni was detected in 83% of samples, followed by As (70%), Hg (50%), Cd (27%), Tl (17%), and Se (13%). We did not detect Ag or Sb in any of the streambed sediment samples. According to the independent t-tests, none of the heavy metal concentrations varied significantly by land cover, but developed sites exhibited higher median concentrations of Pb, Hg, and Zn than undeveloped sites (Table 2). Concentrations of As, Cd, Hg, Ni, Se, and Tl displayed high variability overall, whereas concentrations of Al, Cu, and Zn were considerably less variable (Table 2).

**Table 2:** Summary statistics of all metal concentrations (mg kg^−1^ sediment) detected in streambed sediment samples, grouped by land cover.

Land cover	Statistic	Al	As	Cd	Cr	Cu	Pb	Hg	Ni	Se	Tl	Zn
Developed	Median	17,400	1	0	20	48.7	8.9	0.025	7.8	0	0	80.4
Undeveloped		24,600	1.9	0	20.8	55.4	6.7	0	10.8	0	0	65.5
Developed	Min	13,000	0	0	15.7	27.1	3.4	0	0	0	0	26.7
Undeveloped		12,800	0	0	10.6	31.7	3	0	0	0	0	27.3
Developed	Max	34,600	14.3	1	81.6	82.9	41.2	0.06	79.2	6	1.1	153
Undeveloped		29,600	9.1	0.91	66.2	93	16.1	0.05	60.1	2.9	1.3	95.3
Developed	CV	33.9	166.2	249.3	66.3	31.3	90.4	90.3	172.8	271.3	257.5	44.1
Undeveloped		18.8	90	145.9	56.3	27.2	50.4	137.5	90.5	293	245.3	26.7

None of the metal concentrations varied significantly by land cover (t-test; significance level of 0.05). CV is coefficient of variation.

Out of the 42 VOCs that were tested for, eight were detected in streambed sediments (2-Butanone (MEK), Acetone, Benzene, Ethylbenzene, m&p-Xylene, o-Xylene, Styrene, Toluene). MEK, acetone, styrene, and TPH-GRO were detected only in developed sites, and ethylbenzene and o-xylene were detected only in undeveloped sites (Supplemental Table s-1). Benzene and toluene were the most prevalent VOCs (detected at 83.3% and 60% of sites, respectively) and were detected at both developed and undeveloped sites. MEK, acetone, and TPH-GRO were the most rarely detected VOCs (each present at only one site). None of the VOCs or TPH-GRO concentrations varied significantly by land cover, but maximum VOCs and TPH-GRO concentrations were generally higher at sites within developed areas (Table 3). Mean total organic carbon concentrations (mg kg^−1^ soil) ranged from 2,560 to 141,000 and were higher at sites within developed (mean = 47,684.62), compared to undeveloped areas (mean = 21,896.47; *P* = 0.052; t-test). Soil moisture ranged from 0.99% to 18.1% and was significantly higher at sites within developed (mean = 8.1%), compared to undeveloped sites (3.9%; *P* = 0.018; t-test). We detected significant positive linear relationships between TOC and soil moisture, Cr, Hg, Ni, and Se (Supplemental Table s-2). Mercury was significantly positively correlated with soil moisture, and Cd was significantly positively correlated with Al (Supplemental Table s-2).

**Table 3:** Summary statistics of volatile organic compounds (VOCs) and gasoline range organics (GROs) detected in streambed sediment samples, grouped by land cover.

Land cover	Statistic	2-Butanone (MEK)	Acetone	Benzene	Ethylbenzene	GROs	m&p-Xylene	o-Xylene	Styrene	Toluene
Developed	Median	0	0	11.2	0	0	0	0	0	6.2
Undeveloped		0	0	12	0	0	0	0	0	7.6
Developed	Min	0	0	0	0	0	0	0	0	0
Undeveloped		0	0	0	0	0	0	0	0	0
Developed	Max	0	0	59.7	10	3120	37.2	10.7	0	31.8
Undeveloped		14	18	20.4	0	0	13.4	0	7.6	11.7
Developed	CV	NA	NA	88.5	253	360.6	277.4	360.6	NA	127.1
Undeveloped		412.3	412.3	72.3	NA	NA	412.3	NA	285.9	90

CV is coefficient of variation. None of the concentrations varied significantly by land cover (t-test; significance level of 0.05). Concentrations are in μg kg^−1^ sediment.

### Contamination assessments using geo-accumulation index

We used the Igeo to assess the degree of heavy metal contamination at our sites (Table 4). The Igeo values ranged from 0 to 2.04. According to the Igeo, most (96.7%) samples fell into an uncontaminated category (Igeo 0–1) and 3.3% were moderately contaminated (Igeo = 1–2). Ten percent of sites were moderately contaminated with As, and 23% of sites were moderately contaminated with Cd. Out of the three sites with As Igeo values > 1, two were in developed areas and one was in an undeveloped area. Of the seven sites contaminated with Cd, 71% were in undeveloped areas.

**Table 4:** Summary statistics for geoaccumulation (Igeo) indices of all metals detected in streambed sediment samples, grouped by land cover.

Land cover	Statistic	As Igeo	Cd Igeo	Cr Igeo	Cu Igeo	Pb Igeo	Hg Igeo	Ni Igeo	Se Igeo	Tl Igeo	Zn Igeo
Developed	Median	0.37	0	0.14	0.39	0.08	0	0.15	0	0	0.2
Undeveloped		0.2	0	0.11	0.46	0.07	0.08	0.09	0	0	0.19
Developed	Min	0	0	0.08	0.33	0.04	0	0	0	0	0.16
Undeveloped		0	0	0.06	0.22	0.03	0	0	0	0	0.08
Developed	Max	1.91	1.66	0.47	0.57	0.41	0.24	0.79	0.02	0.02	0.43
Undeveloped		1.22	2.05	0.14	0.75	0.16	0.21	0.16	0.01	0.02	0.27
Developed	CV	114.6	244.4	64.1	19.9	101.7	128.9	108.3	285.6	210.6	36.4
Undeveloped		123.4	147.3	17.5	34.2	55.3	105.5	59.4	284.4	297.2	36.1

Only the Igeo for chromium varied significantly by land cover and was higher at developed, compared to undeveloped sites (P = .045). CV is coefficient of variation.

### Principal component analyses

#### Principal component analyses to assess drivers of spatial variability

We used PCA to test for underlying patterns in contaminant concentrations according to land cover, soil order, TOC, elevation, distance to road, and distance to shore, but we did not detect any clear patterns in the metals or volatiles concentrations according to these parameters (Supplemental Figs. s-1 and s-2).

#### Metals PCA

**Figure 2:**
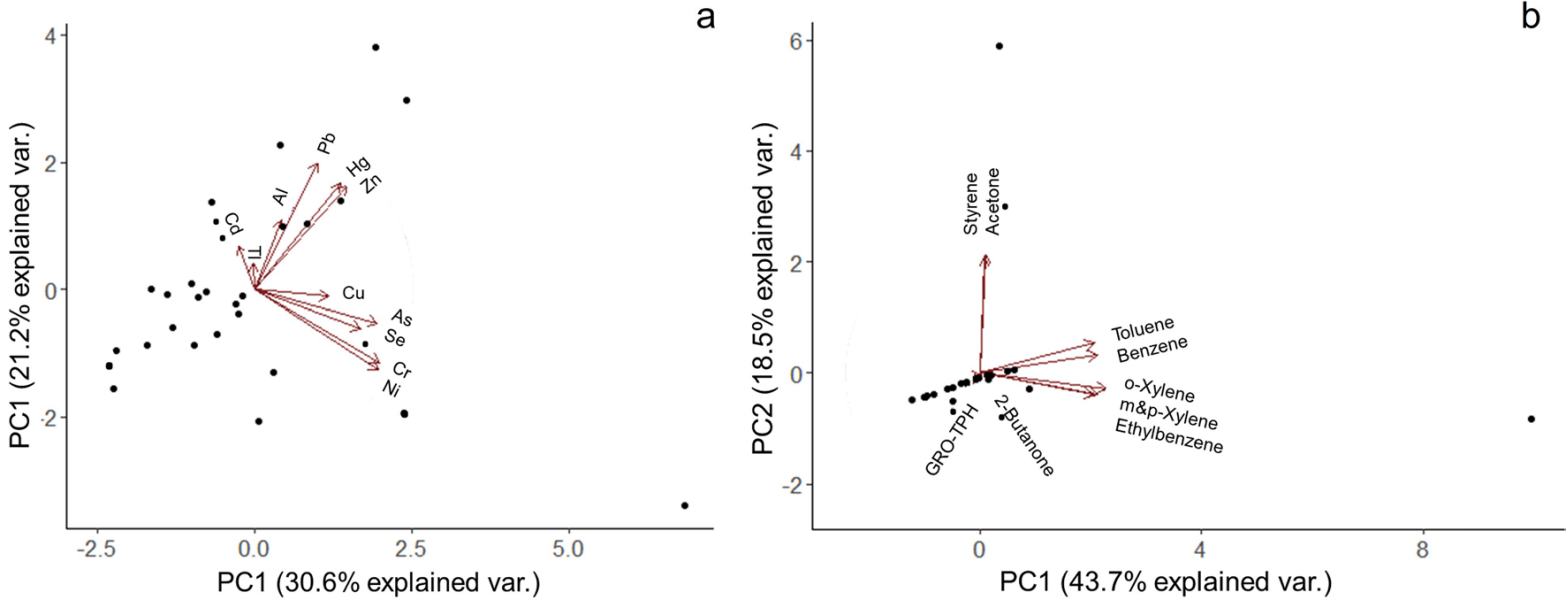
Principal component analysis (PCA) biplots displaying PCA scores of the first (x-axis) and the second (y-axis) principal components of the metals PCA (**a**) and volatiles PCA (**b**). The explanatory variables are represented with red vectors and the individual observations (N = 30) are displayed as black circles.

**Figure 3:**
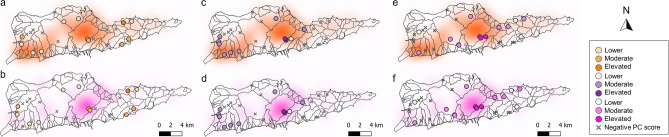
Positive principal component (PC) 1 (**a** and **b**), PC2 (**c** and **d**), and PC3 (**e** and **f**) scores from the metals PCA overlaid on heatmaps of residential (**a**,**c**,**e**) and commercial (**b**,**d**,**f**) buildings. Positive PC values are color-coded according to groups (lower-elevated) that correspond to their magnitude. Watershed boundaries are shown as gray lines.

**Figure 4:**
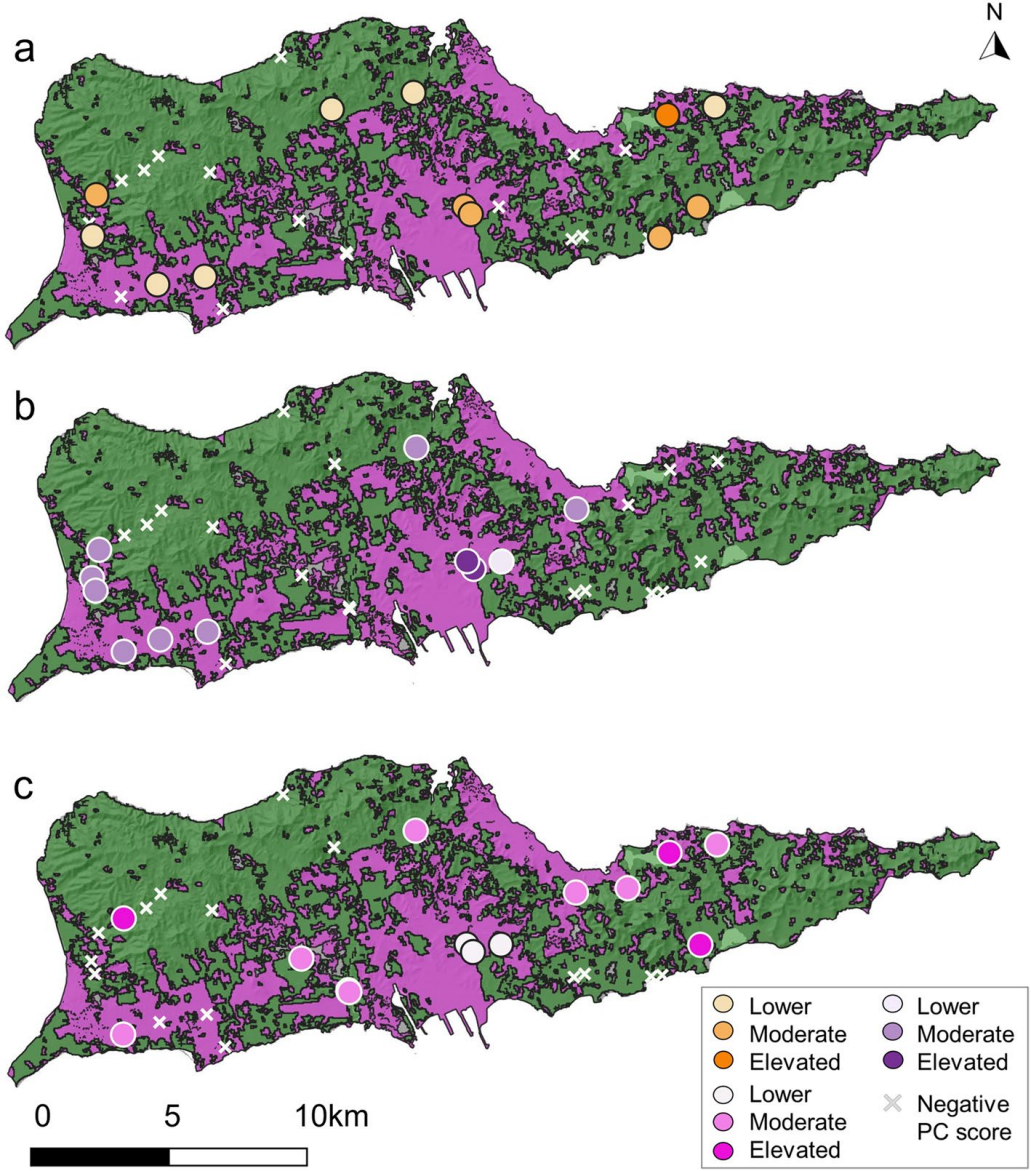
Maps of St. Croix, USVI with developed (magenta) and undeveloped (green) land cover classifications. Principal component (PC) scores from (**a**) PC1, (**b**) PC2, and (**c**) PC3 of the metals principal component analysis (PCA) are color-coded according to their magnitude. Map created using QGIS software, version 3.28.11, https://www.qgis.org/en/site/forusers/download.html.

We performed separate PCAs on correlation matrices of (i) all metal concentrations (hereafter referred to as the “metals PCA”; Table 5) and (ii) VOCs and TPH-GRO concentrations (hereafter referred to as the “volatiles PCA”; Table 6). The PCA placed the heavy metals into three main groups (Fig. 2a): Cr, Ni, As, and Se (accounting for 57% of PC1); Pb, Hg, and Zn (accounting for 47% of PC2); Al, Cd and Tl (accounting for 55% of PC3). Of the 12 sites with positive PC1 scores (i.e., relatively high Cr, Ni, As, and Se), 50% were in developed areas. Except for four sites on the east end of the island, sites with positive PC1 scores were generally in proximity to areas with high residential building density (Fig. 3a) and were concentrated close to the coast on mostly flat terrain (Fig. 4a). Only some sites with positive PC1 scores were near areas with high commercial building densities (Fig. 3b). Results of Pearson correlation analyses indicated that positive PC1 scores were not significantly correlated with residential (*P* = 0.938; r = − 0.025) or commercial building densities (*P* = 0.743; r = 0.106).

**Table 5:** Principal component (PC) loadings for the first three PCs of the metals principal component analysis (PCA).

Component	PC1 *30.6%*	PC2 *21.2%*	PC3 *15.6%*
Al	0.095	0.282	− 0.542
As	0.424	− 0.136	− 0.037
Cd	− 0.055	0.18	− 0.511
Cr	0.43	− 0.299	0.105
Cu	0.257	− 0.028	− 0.302
Pb	0.217	0.518	0.273
Hg	0.297	0.432	− 0.103
Ni	0.429	− 0.327	− 0.055
Se	0.368	− 0.163	− 0.153
Tl	− 0.003	0.107	− 0.44
Zn	0.32	0.424	0.19

Variability explained by each PC is italicized.

**Table 6:** Principal component (PC) loadings for the first two PCs of the volatiles principal component analysis (PCA).

Component	PC1 *43.7%*	PC2 *18.5%*
2-Butanone	0.008	− 0.0137
Acetone	0.017	0.668
Benzene	0.444	0.107
Ethylbenzene	0.43	− 0.124
m&p-Xylene	0.445	− 0.121
o-Xylene	0.478	− 0.093
Styrene	0.022	0.685
Toluene	0.434	0.176
TPH-GRO	− 0.023	− 0.058

Variability explained by each PC is italicized.

The majority (64%) of sites with positive PC2 scores (i.e., elevated Pb, Hg, and Zn concentrations) were in developed areas (Fig. 4b). Sites with positive PC2 scores appeared to exhibit a strong spatial pattern consistent with commercial building density (Fig. 3d), and we detected a significant positive Pearson correlation between positive PC2 scores and commercial building density (*P* = 0.013; r = 0.72). In contrast, we did not detect a significant correlation between positive PC2 scores and residential building density (Fig. 3c; Pearson correlation; *P* = 0.347; r = 0.314). When the first two PCs were plotted and color-coded according to land cover, soil properties, and spatial characteristics, the observations did not cluster into distinct groups (Supplemental Fig. s-2).

PC3 can uniquely be characterized as lacking Al, Cd, and Tl, as these metals had highly negative loadings. Approximately half (53%) of sites lacking Al, Cd, and Tl were in undeveloped areas (Fig. 4c). The remaining 47% of sites were developed and were close to commercial buildings (Fig. 3f). Some sites with positive PC3 scores were in areas of high residential building density, but we did not observe the same strong spatial pattern as with commercial buildings (Fig. 3e and f). Pearson correlation analysis indicated that positive PC3 scores were significantly correlated with commercial (*P* = 0.004; r = 0.89), but not residential (*P* = 0.319; r = 0.276) building densities.

### Volatiles PCA

**Figure 5:**
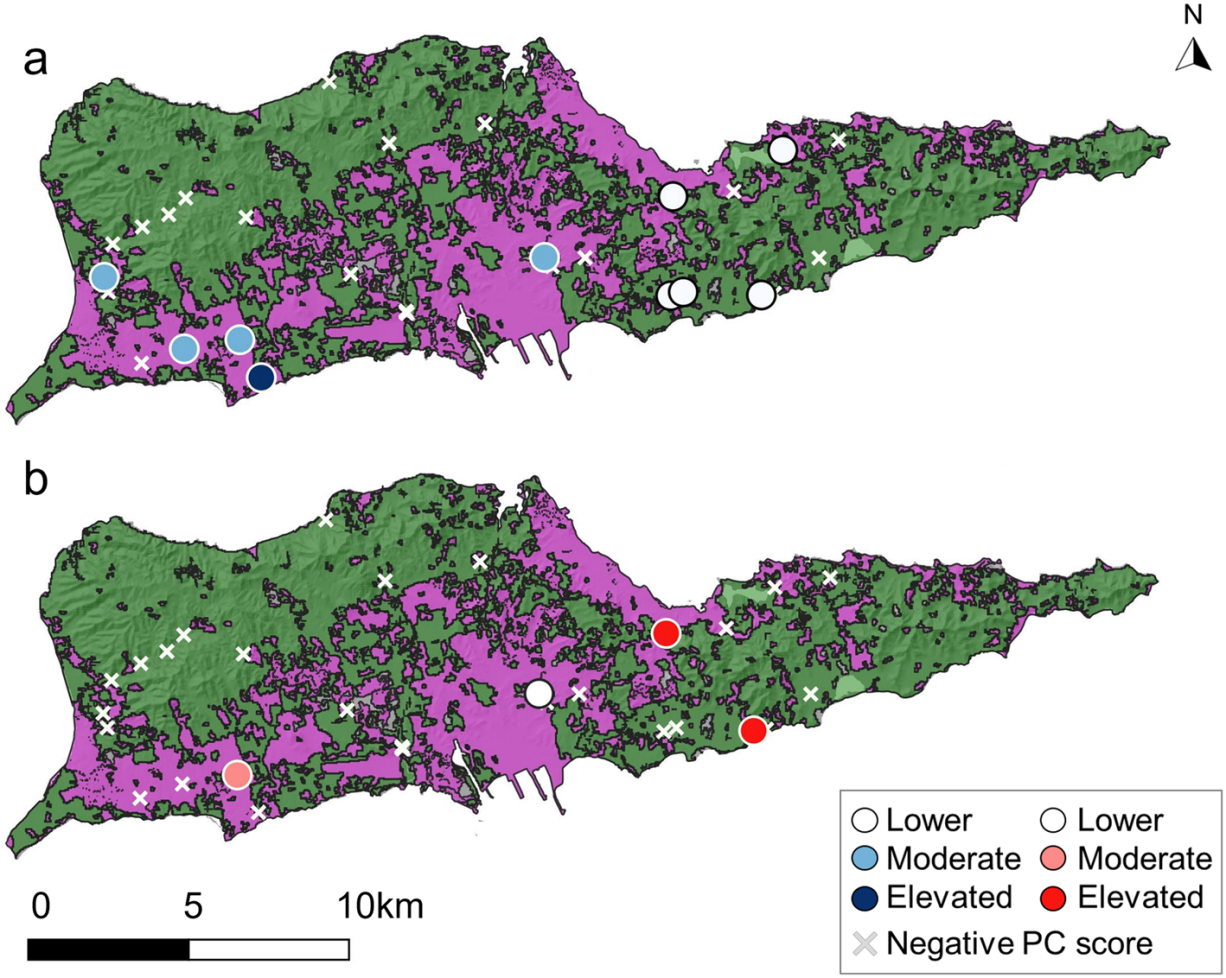
Maps of St. Croix, USVI with developed (magenta) and undeveloped (green) land cover classifications. Principal component (PC) scores from (**a**) PC1 and (**b**) PC2 of the volatiles principal component analysis (PCA) color-coded according to their magnitude. Map created using QGIS software, version 3.28.11, https://www.qgis.org/en/site/forusers/download.html.

**Figure 6:**
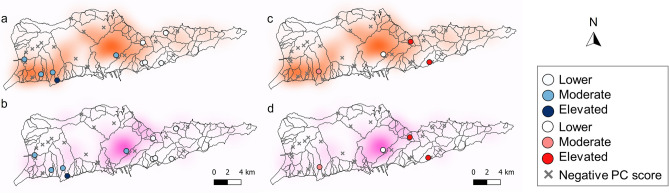
Positive principal component (PC) 1 (**a** and **b**) and PC2 (**c** and **d**) scores from the volatiles PCA overlaid on heatmaps of residential (**a** and **c**) and commercial (**b** and **d**) buildings. Positive PC values are color-coded according to groups (lower-elevated) that correspond to their magnitude. Watershed boundaries are shown as gray lines. Map created using QGIS software, version 3.28.11, https://www.qgis.org/en/site/forusers/download.html.

The first two PCs of the volatiles PCA had eigenvalues > 1 and cumulatively accounted for 62% of the variability in the dataset (Table 6). The PCA placed the VOCs into two distinct groups: o-xylene, m&p-xylene, benzene, toluene, and ethylbenzene (accounting for 99% of PC1), and styrene and acetone (accounting for 66% of PC2). When scores of the first two PCs were plotted, they clustered tightly into one group, except for three sites with markedly higher volatiles concentrations (Fig. 2b). The majority (70%) of sites with positive PC1 scores (i.e., high concentrations of o-xylene, m&p-xylene, benzene, toluene, and ethylbenzene) were in undeveloped areas. Most sites with positive PC1 scores were on the south shore of the island, except for two sites on the north shore (Fig. 5a). Positive PC1 scores were not significantly correlated with residential (Fig. 6a; *P* = 0.775; r =  − 0.104; Pearson correlation) or commercial building densities (Fig. 6b; *P* = 0.853; r = 0.067; Pearson correlation). Half of the sites with positive PC2 scores (i.e., high styrene and acetone concentrations) were in developed areas (Fig. 5b). Most sites with positive PC2 scores were in areas of high residential and commercial building densities (Fig. 6c and d), but positive PC2 scores were not significantly correlated with residential (*P* = 0.498; r =  − 0.502; Pearson correlation) or commercial (*P* = 0.905; r =  − 0.095; Pearson correlation) building densities. The observations did not cluster into distinct groups when the first two PCs were plotted and color-coded according to spatial and soil properties (Supplemental Fig. s-2).

## Discussion

We measured streambed sediment heavy metals and VOCs on St. Croix, USVI to assess chemical pollution within the island’s dry intermittent streambeds. Contamination was generally concentrated in flat, lower-lying areas that are close to the coast and undergo increased human activity. These lower-lying nearshore sites are also downstream of larger catchment areas. The convergence of surface runoff and streamflow at the watershed’s outflow points may have resulted in higher pollutant transport to these areas. In contrast to our hypothesis, pollutant concentrations did not vary significantly by land cover (Figs. 4 and 5). Results of the PCAs also revealed that the pollutant concentrations did not exhibit a clear spatial pattern based solely on land cover classification (Supplemental Figs. s-1 and s-2). This finding agrees with those of Aelion and others^66^ and Davis and others^62^, who detected heavy metal pollution in both urban and rural areas.

**Figure 7:**
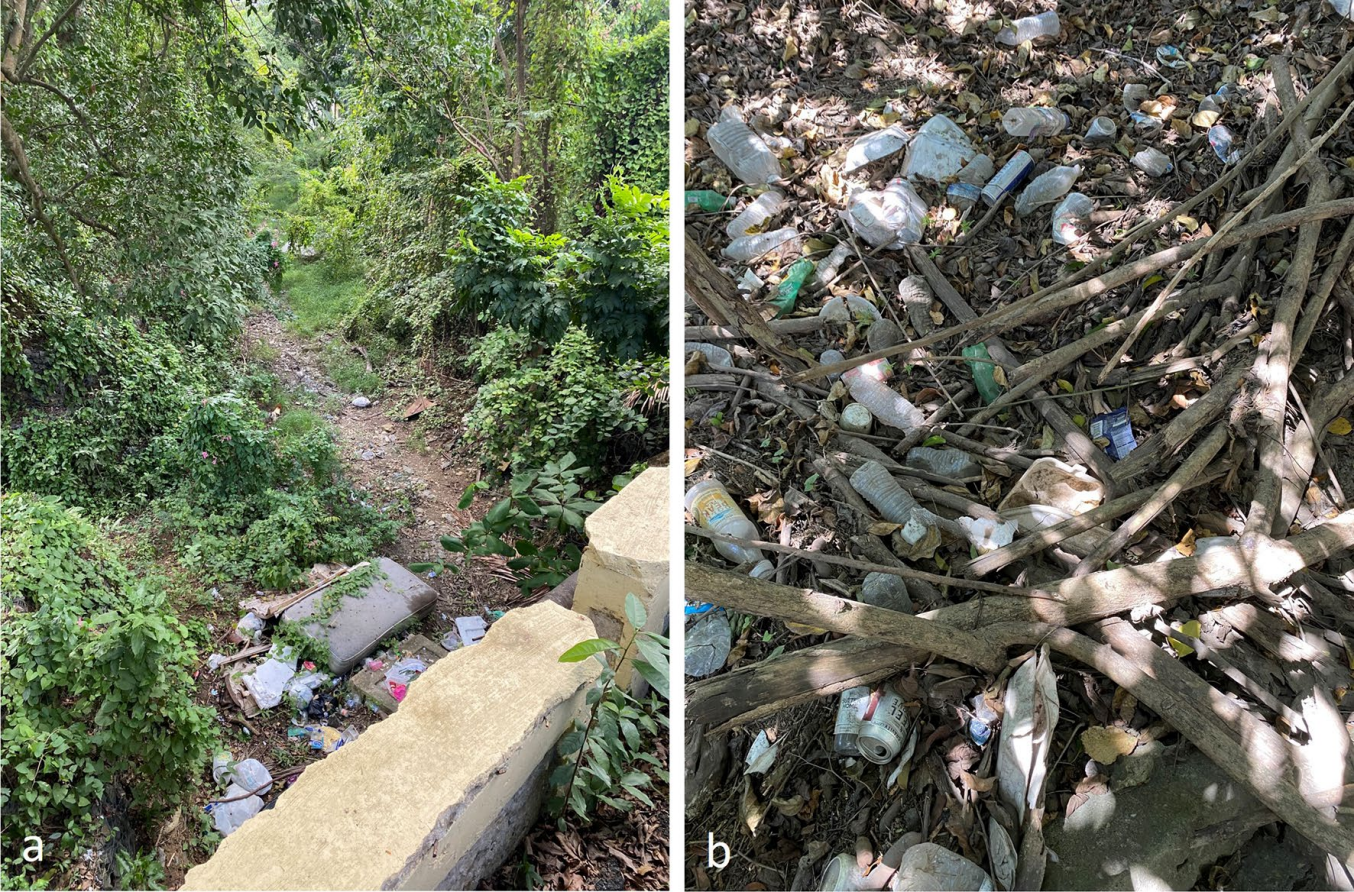
Photographs of improperly discarded waste in intermittent streambeds (locally referred to as guts) located in Christiansted (**a**) and Frederiksted (**b**), St. Croix. Photograph (**a**) was captured from above, on a bridge, and shows a discarded mattress and other debris. Photograph (**b**) was taken close to the ground to show discarded cans, bottles, and food containers among sediment, leaves, and branches.

The proximity of our study sites to roads could explain elevated pollutant concentrations in undeveloped areas. Accessibility was a requirement for site selection, and all undeveloped sites were within 220 m of a vehicle-accessible road (Table 1). Both exhaust and non-exhaust (i.e., wear of tires, brakes, clutch plates, road surfaces) vehicle emissions are known contributors of heavy metal pollution^67^. Additionally, runoff from paved or unpaved roads can transport pollutants to streambeds^68^. Illegal dumping could also contribute to the widespread presence of heavy metals and VOCs. Due to inadequate solid waste management within the territory, illegal dumping in intermittent streambeds occurs frequently^13^. Improperly discarded electronics, household goods, and automotive parts found in the stream channel and streambank at some of our study sites could therefore be sources of heavy metals and VOCs^69–71^. It should be noted that severe flooding and high streamflow during tropical storms and hurricanes can also mobilize household debris to streambeds. Contamination and the presence of solid waste in the streambeds (Fig. 7) highlights a need for increased community awareness of illegal dumping, as well as practical, stakeholder-supported strategies to improve USVI waste management and reduce improper waste disposal.

More than half of our undeveloped sites are located within 350 m of residential properties, and about 40–60% of St. Croix’s population relies on poorly to moderately functioning septic systems^72^. Septic tank sewage sludge can contain Pb, Hg, and Zn, and corrosion of outdated Cu and Pb pipes can contaminate wastewater effluent^73^. Poor soil suitability and outdated wastewater treatment infrastructure throughout the territory are well-documented, suggesting septic system failures are commonplace and may contribute to streambed sediment contamination^72^.

We did not detect clear spatial patterns of heavy metals or VOCs according to land cover, soil order, TOC, elevation, distance to road, or distance to shore. The contaminant concentrations did not exhibit any robust spatial patterns consistent with residential building density, but we did detect a strong spatial pattern in Pb, Hg, and Zn levels (PC2 of the metals PCA) according to commercial building density. The highest levels of Pb, Hg, and Zn were concentrated within a hotspot of commercial buildings, suggesting the presence of these heavy metals resulted from increased anthropogenic activity in this area. Our finding agrees with those of Li and others^74^ who sampled soils within commercial, residential, and industrial areas of Hong Kong and found concentrations of Cd, Cu, Pb and Zn to be highest in commercial areas. Similarly, Kamani and others^75^ observed a strong pollution signal from commercial areas in Iran, as the degree of heavy metal pollution in street dust was highest in commercial areas compared to urban parks and residential, industrial, and high traffic areas.

The PCAs identified distinct sub-groups of heavy metals and volatiles (Fig. 2). Co-occurrence of contaminants could indicate a shared pollution source or chemical affinities of certain metals or volatiles, thus resulting in their coexistence in the environment^76^. Results from the metals PCA grouped Cr, Ni, As, and Se as PC1. We did not observe a clear spatial pattern that properly characterized all sites with elevated Cr, Ni, As, and Se (Supplemental Fig. s-2). Sites with elevated Cr, Ni, As, and Se were evenly distributed among developed and undeveloped land cover classes (Fig. 4a).

We identified anthropogenic hotspots that contextualize the co-occurrence of these metals and suggest their presence originated from unnatural sources. For example, two sites with elevated Cr, Ni, As, and Se are in St. Croix’s second largest watershed (Hovensa, 19.7 km^2^), which is highly developed. These sites are in the vicinity of heavy industry and are located downstream of an expansive impervious area, including a large shopping plaza highly congested with vehicle traffic, automotive repair shops, a parking lot storing old trucking equipment, a tire and car battery retailer, etc. Other sites within this group are adjacent to a heavily trafficked road. Automotive parts and industrial, commercial, or residential fuel combustion are known sources of Cr, Ni, As, and Se pollution^69^.

Maximum levels of Cr, Ni, As, and Se were detected at a site that receives runoff from a golf course and a frequently flooded parking lot that contains construction vehicles. Phosphate fertilizer contains Cr and Ni and can be used to fertilize golf courses^77^. We observed maximum soil moisture and TOC content at this site, which has frequently flooded soils that are classified as Mollisols (i.e., having high organic content)^50^. High carbon content typically increases the mobility of heavy metals within soil, which agrees with this observation and could explain maximum Cr, Ni, As, and Se found here^78,79^. As high leaching rates pose significant risks to groundwater quality^80^, streambed sediments on St. Croix that have elevated TOC and are in proximity to contamination sources should be monitored regularly.

Sites with elevated PC3 scores of the metals PCA can be characterized as lacking Al, Cd, and Tl, as these metals had highly negative loadings. Cadmium and Tl were undetectable at most sites, and this spatial pattern likely drove PCA clustering. We detected strong correlations between Al and Cd (Supplemental Table s-2), suggesting Cd had a lithogenic origin at most sites^20,81^. Seven sites (all in developed areas) had unnatural (i.e., “moderately polluted) Igeo values for Cd. All sites are in proximity to roads, and some are close to agricultural land.

Xylenes, benzene, and toluene were grouped into PC1 of the volatiles PCA (Fig. 2b) and are collectively referred to as ‘BTEX’ because of their closely related chemical structures. These VOCs have similar fate and transport properties and co-occur in the environment as pollutants^69^. BTEX are components of crude oil and can contaminate soil via air emissions from vehicle exhaust or the oil refining process, or from gasoline leaks or spills^69^. Some sites with BTEX were on the south shore of St. Croix (where industrial activities are concentrated), including a site that is approximately 1.6 km downwind of a crude oil refinery that operated for almost four decades. Streambed sediments in proximity to this area could be contaminated with VOCs released from industrial emissions. Benzene and other VOCs were detected during an air quality monitoring campaign on St. Croix’s south shore in 2011^82^. However, the process of oil refining has been shown to emit a suite of VOCs that were not measured in the present study (e.g., 2-methylhexane, 2,3-dime-thylpentane, 2,4-dimethylpentane, cyclohexane, 3-methylhexane, 2-methylheptane, n-heptane, and methylcyclohexane)^83^. Therefore, additional research that includes sampling the area of interest for these VOCs with higher spatial resolution, and subsequent source apportionment using a receptor model (e.g., Positive Matrix Factorization model), is therefore required to determine the origin of the contamination detected.

Other sites with BTEX were adjacent to heavily trafficked roads on the north and south shores of the island. Several apportionment studies, including one conducted in Texas, USA in the vicinity of an operational oil refinery^83^, show that the co-occurrence of xylenes, benzene, and toluene in air is strongly linked to vehicle exhaust. One site on the south shore had a disproportionately high PC1 score (Fig. 2b). Because VOCs can volatilize quickly after they are deposited onto soil^84^, this site was most likely contaminated by a recent, localized gasoline leak or spill.

Styrene and acetone were found to co-occur at our sites and were clustered together by the volatiles PCA into PC2. The highest levels of styrene and acetone were detected at two sites that are adjacent to heavily trafficked roads. Styrene and acetone are used to manufacture plastics and can be found in consumer products that are commonly littered, such as disposable packaging. Acetone can also be emitted to the atmosphere via vehicle exhaust^69^. This distinctive group of VOCs may therefore represent contamination from both improper solid waste disposal and vehicle exhaust.

Mean concentrations of Cu, Ni, Cr, and Cd found in the present study exceeded the range of concentrations detected in streambed sediment collected during the dry season in southern Nigeria^37^. Chromium concentrations detected in the present study exceeded those recorded by Iwegbue and others by two orders of magnitude, whereas Pb and Zn concentrations were within the range they reported^37^. Mean concentrations of select heavy metals detected in the present study were similar to mean concentrations found in streambed sediments within a heavily industrialized watershed in China (Cd = 0.29 mg kg soil^−1^, Cr = 30, Cu = 34, Ni = 23, Zn = 71)^85^. In a separate study, mean Cd and Zn concentrations reported for sediment within a reservoir surrounded by industrial and agricultural land use in China were approximately four times higher than those detected in the present study^86^. In Southeast Asia, Zarcinas and others^87^ reported mean concentrations of Pb and As in agricultural inceptisols (33.4 and 9.4 mg kg soil^−1^, respectively) that were higher than those detected in our study. Mean Cd and Cu concentrations from the current study were approximately twice as high as the levels reported by Zarcinas and others^87^.

Concentrations of anthropogenic VOCs in indoor and outdoor air are regulated and well-documented, but data on soil anthropogenic VOC levels are limited. We detected ethylbenzene and styrene at only a few sites, but due to their potentially carcinogenic nature, the presence of ethylbenzene and styrene in the environment is of concern to human and ecological health. Ethylbenzene and styrene are rapidly degraded in soil and are thus rarely detected in the soil environment, indicating their presence at our sites is likely a result of recent contamination. Recent data on levels of toluene, 2-butanone, or xylenes are scarcely available^69^. Similarly, the presence of 2-butanone and xylenes in soil is rare but concerning for human and ecological health. Benzene concentrations from the present study (0 to 59.7 ug kg^−1^) were within the range of levels reported for soils in proximity to industrial production or utilization of benzene (2–191 ug kg^−1^)^69^ but were considerably lower than levels detected in metropolitan areas of Detroit, MI, USA (7.9 × 106 ug kg^−1^)^88^. The median concentration of benzene detected in the present study (11.6 ug kg^−1^) is more than twice the median level of benzene detected in soils within the contiguous United States^69^. Acetone was detected at only one site, and the concentration was three times that of non-hazardous levels^69^.

Arsenic and Cd were the only heavy metals detected at “moderately polluted” Igeo levels. The rest of the heavy metals were detected at “unpolluted to moderately polluted” levels. Most sites with moderately polluted Igeo levels for Cd were in undeveloped areas, whereas sites with moderately polluted Igeo levels for As were in both developed and undeveloped areas. We compared streambed sediment heavy metal concentrations detected in our study to guidelines established by the USEPA for As, Cd, Cr, Cu, Ni, Pb, and Zn in freshwater sediment^89^ and permissible levels of Cd, Cr, Cu, Ni, and Pb in soil according to the World Health Organization (WHO)^90^ (Supplemental Table s-3). Arsenic exceeded USEPA guidelines for freshwater sediment at two sites, which were in developed areas. Cadmium concentrations exceeded USEPA limits at seven sites (two developed, five undeveloped) and WHO limits at five sites (one developed, four undeveloped). Chromium exceeded USEPA limits for freshwater sediment at four sites (one developed, three undeveloped) but did not exceed WHO permissible soil levels. At most sites, Cu concentrations detected in the present study exceeded limits established by USEPA (97% of sites) and WHO (83% of sites). Mercury levels did not exceed the mean soil Hg concentration for the contiguous United States (0.08 mg kg^−1^)^69^. Nickel concentrations at six sites exceeded the USEPA limit (five developed, one undeveloped) and the WHO limit at three sites (one developed; two undeveloped). Lead exceeded the USEPA limit at only one site, which was in a developed area. However, Pb concentrations were within the WHO permissible limit at all sites. At two sites (both developed) Zn levels exceeded the USEPA limit. Thallium levels at two sites (one developed, one undeveloped) exceeded the environmentally safe limit of 1 mg kg^−1^ soil^91,92^. Selenium concentrations were within the range of safe levels (0–5 mg kg^−1^ soil)^93^.

These findings further indicate that the presence of heavy metals within our study area did not follow a clear spatial pattern based only on land cover. As most samples were characterized as unpolluted by the Igeo, there was poor agreement between the Igeo metric and contamination levels determined using toxicity regulations. Our results show that the method to determine soil heavy metal contamination levels should be chosen based on the specific data application. Overall, the detection of contaminants in both developed and undeveloped areas suggests that land use (not available for the USVI when the present study was conducted), which is a specific indicator of human activity, may serve as a more accurate indicator of contaminant spatial distribution than land cover.

Furthermore, additional research is required for a more detailed characterization of human impact on intermittent streams within our study area. Enhancing the spatial resolution of our dataset through additional island-wide sampling, paired with an interpolation method (e.g., kriging), could improve our ability to identify or confirm contamination sources^63^. By expanding our analysis to include contaminant concentrations in soils beyond intermittent streambeds, more intricate spatial patterns could be revealed. For example, additional data are needed to distinguish between legacy pollution from industrial activities and recent, localized pollution from other sources. Further sampling and analysis at various soil depths could be used to assess changes in pollutant levels over time and to distinguish between background contamination (deeper depths) and more recent, anthropogenic contamination (shallower depths)^94^.

## Conclusion

Streambed sediment contaminant concentrations measured on St. Croix, USVI did not follow a strict land cover pattern. Elevated heavy metal and VOC levels were generally concentrated on the perimeter of the island, in flat areas, where most human-land interactions occur, and where surface runoff and streamflow converge. Concentrations of Pb, Hg, and Zn were correlated with commercial building density, suggesting their presence resulted from anthropogenic activity. The PCA results pointed to other potential pollution sources, including automotive parts and fuel combustion (Cr, Ni, As, and Se), vehicle exhaust, oil refining, and gasoline leaks (2-butanone and xylenes), and plastics (acetone and styrene). The most prevalent VOCs were benzene and toluene and were found at both developed and undeveloped sites. At some sites, select heavy metal concentrations (e.g., As, Cd, Cr, Ni) exceeded USEPA and/or WHO guidelines. This study serves as a baseline assessment of anthropogenic impact on intermittent streams on St. Croix, USVI. As the first of their kind, the results can be shared with government agencies, community members, and other scientists to bring awareness to the pollution issue. Especially as human development continues to expand rapidly throughout the USVI, continued assessments of anthropogenic impact on intermittent streams is critical.

### Supplementary Information

Supplementary Information.

## Data Availability

The datasets generated and analyzed for the current study are available in the Zenodo Data Repository under Creative Commons license via https://zenodo.org/record/7620708#.Y-Ogz3bMJD8 without a registration requirement.
