# Supplementary material for: Detection of heavy metals and VOCs in streambed sediment indicates anthropogenic impact on intermittent streams of the U.S. Virgin Islands
Source: Sci Rep. 2023 Oct 11;13:17238. doi: 10.1038/s41598-023-44455-2 (PMC10567703; doi:10.1038/s41598-023-44455-2)
Supplement: Supplementary file 1 — Supplementary Information. [file 41598_2023_44455_MOESM1_ESM.docx]

Supplemental Material

**Detection of heavy metals and VOCs in streambed sediment indicates anthropogenic impact on intermittent streams of the U.S. Virgin Islands**

Brittany V. Lancellotti^1*^, David A. Hensley^1,2^ and Race Stryker^2^

^1^Virgin Islands EPSCoR, University of the Virgin Islands, Kingshill, VI 00850

^2^Agricultural Experiment Station, University of the Virgin Islands, Kingshill, VI 00850

*Corresponding author: Brittany Lancellotti ([Brittany.lancellotti@uvi.edu](mailto:Brittany.lancellotti@uvi.edu))

This supplemental document contains six pages of three tables and two figures. The supplemental materials provide results of simple linear regression analyses that assess relationships between contaminants and soil conditions. Also available in this document are supplemental materials providing a comparison of regulatory heavy metals limits to concentrations detected in the present study and biplots resulting from the principal components analysis that are color-coded according to several explanatory variables.

- Supplemental Table s-1 provides a list of all volatile organic compounds tested for in the study and indicates if they were detected at the study sites
- Supplemental Tables s-2 provides statistically significant linear relationships between pollutants and soil properties using simple linear regression
- Supplemental Table s-3 provides regulatory concentrations for heavy metals in soil or freshwater sediment
- Supplemental Figures s-1 and s-2 display principal components color-coded by various properties that were expected to explain variability in contaminant concentrations

| **Supplemental Table s-1** List of volatile organic compounds that all 30 streambed sediment samples were sampled for. D = analyte detected in at least one sample; ND = analyte not detected in any samples | | |
| --- | --- | --- |
| Volatile organic compound | Developed | Undeveloped |
| 1,1-Dichloroethane | ND | ND |
| 1,1-Dichloroethene | ND | ND |
| 1,1,1-Trichloroethane | ND | ND |
| 1,1,2-Trichloroethane | ND | ND |
| 1,1,2,2-Tetrachloroethane | ND | ND |
| 1,2-Dibromo-3-chloropropane | ND | ND |
| 1,2-Dibromoethane (EDB) | ND | ND |
| 1,2-Dichloroethane | ND | ND |
| 1,2-Dichloropropane | ND | ND |
| 2-Butanone (MEK) | ND | D |
| 2-Hexanone | ND | ND |
| 4-Methyl-2-pentanone | ND | ND |
| Acetone | ND | D |
| Benzene | D | D |
| Bromodichloromethane | ND | ND |
| Bromoform | ND | ND |
| Bromomethane | ND | ND |
| Carbon disulfide | ND | ND |
| Carbon tetrachloride | ND | ND |
| Chlorobenzene | ND | ND |
| Chloroethane | ND | ND |
| Chloroform | ND | ND |
| Chloromethane | ND | ND |
| cis-1,2-Dichloroethene | ND | ND |
| cis-1,3-Dichloropropene | ND | ND |
| Dibromochloromethane | ND | ND |
| Dichlorodifluoromethane | ND | ND |
| Ethylbenzene | D | ND |
| Isopropylbenzene (Cumene) | ND | ND |
| m&p-Xylene | D | D |
| Methyl acetate | ND | ND |
| Methyl-tert-butyl ether | ND | ND |
| Methylene Chloride | ND | ND |
| o-Xylene | D | ND |
| Styrene | ND | D |
| Tetrachloroethene | ND | ND |
| Toluene | D | D |
| trans-1,2-Dichloroethene | ND | ND |
| trans-1,3-Dichloropropene | ND | ND |
| Trichloroethene | ND | ND |
| Trichlorofluoromethane | ND | ND |
| Vinyl chloride | ND | ND |

| **Supplemental Table s-2** Significant (*P* < 0.05) linear relationships detected between pollutants and soil properties when simple linear regression analyses were performed including all observations (n=30) | | | | |
| --- | --- | --- | --- | --- |
| Dependent variable | Independent variable | *p* value | R-squared | F-statistic |
| Cadmium | Aluminum | 0.007 | 0.21 | 8.48 |
| Chromium | TOC | 0.017 | 0.19 | 6.43 |
| Mercury | TOC | 0.002 | 0.26 | 11.18 |
| Nickel | TOC | 0.038 | 0.11 | 4.76 |
| TOC | Soil moisture | <0.001 | 0.43 | 22.46 |
| Selenium | TOC | 0.002 | 0.28 | 12.01 |
| Mercury | Soil moisture | 0.032 | 0.123 | 5.08 |

| **Supplemental Table s-3** Regulatory concentrations for heavy metals in soil or freshwater sediment. All values reported in mg kg ^-1^ | | | | |
| --- | --- | --- | --- | --- |
|  | Concentration ranges from current study | Mean soil concentration or typical range of soil concentrations in contiguous U.S.  (ATSDR, 2003) | USEPA guidelines for freshwater sediment  (USEPA, 1994) | Permissible levels of heavy metals in soil (WHO, 1996) |
| As | 0 – 14.3 | 7.2 | 10.798 | N/A |
| Cr | 10.6 – 81.6 | 37 | 36.286 | 100 |
| Hg | 0 – 0.059 | 0.08 | N/A | N/A |
| Ni | 0 – 79.2 | 4 – 80 | 19.514 | 35 |
| Pb | 3 – 41.2 | 15 – 20 | 37.000 | 85 |
| Se | 0 – 6 | 0.1 – 0.2 | N/A | N/A |
| Zn | 26.7 – 153 | 60 | 98.000 | N/A |
| Cd | 0 – 1 | 0.06 – 1.1 | 0.583 | 0.8 |
| Cu | 27.1 – 93 | 0.001 – 150,000 | 28.012 | 36 |

**Figure s-1** Positive principal component (PC) 1 (a and b), PC2 (c and d), and PC3 (e and f) scores from the metals PCA overlayed on heatmaps of residential (a, c, and e) and commercial (b, d, and f) buildings. Positive PC values are color-coded according to groups (lower-elevated) that correspond to their magnitude. Watershed boundaries are shown as black lines


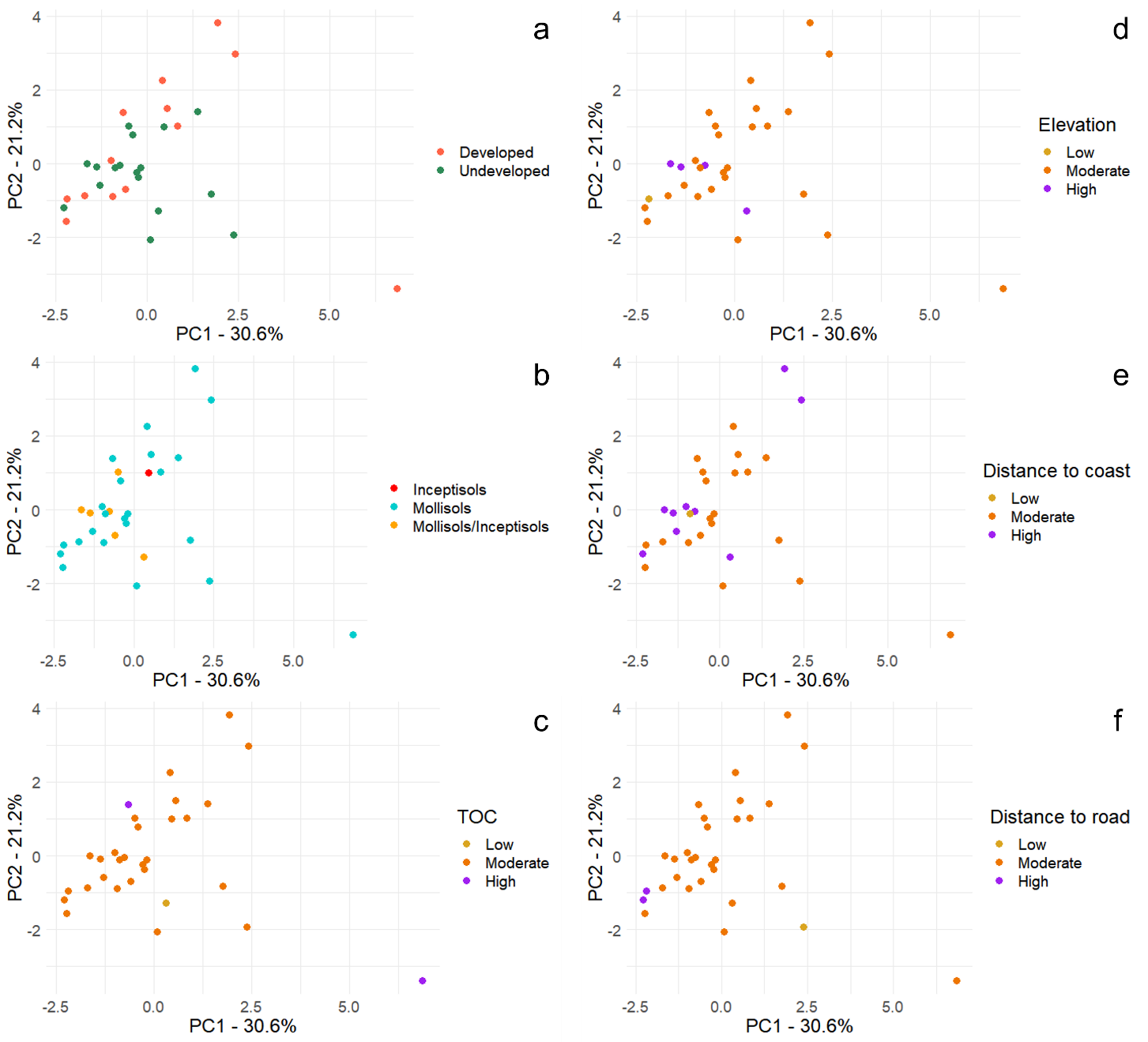


**Supplemental Figure s-1** Principal component 1 (x-axis) and principal component 2 (y-axis) of the metals principal component analysis (PCA) color-coded by (a) land cover, (b) soil order, and categories representing the magnitude (low-high) of (c) total organic carbon (TOC), (d) elevation (e) distance from site to nearest coast, and (f) distance from site to nearest road


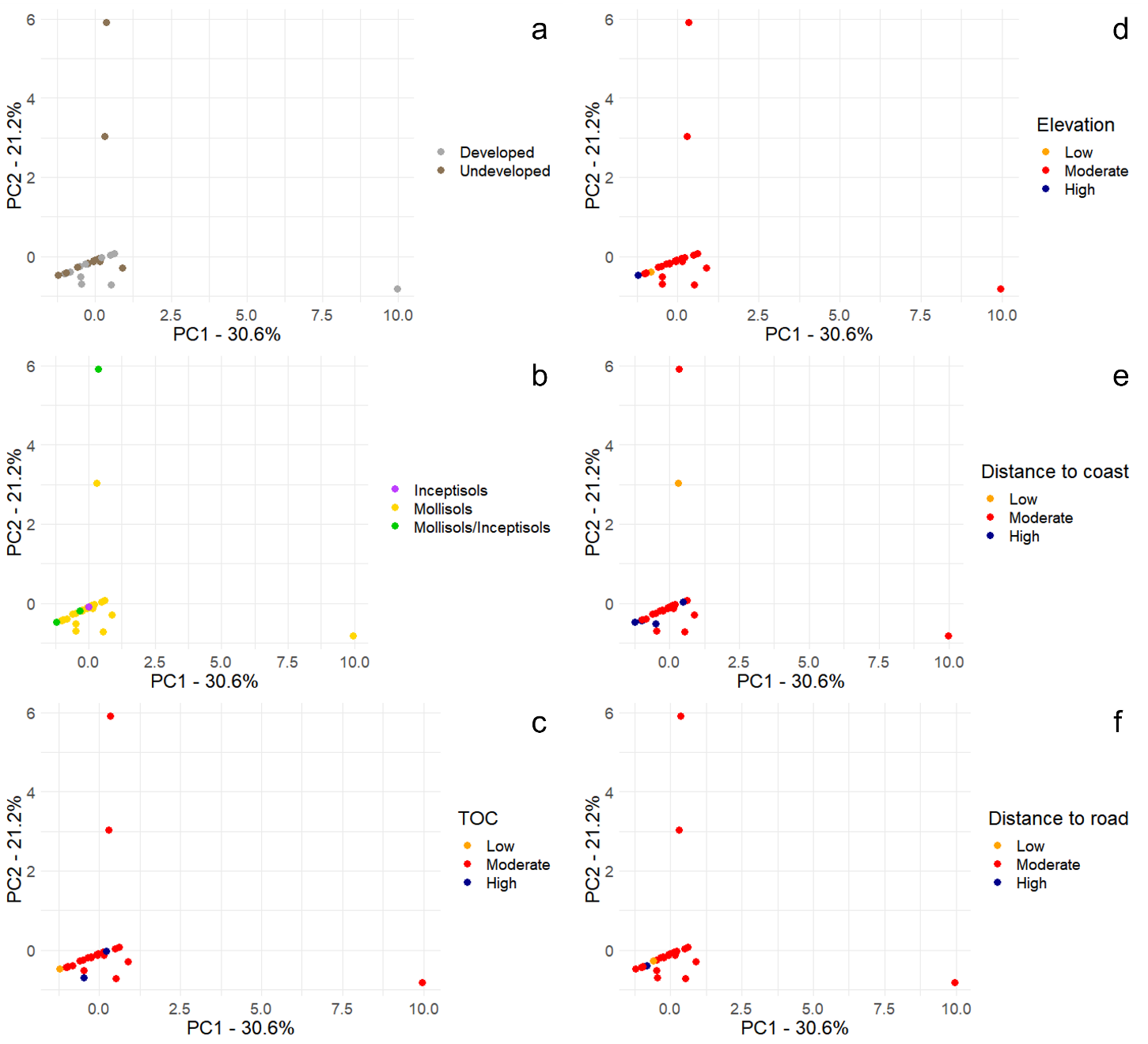


**Supplemental Figure s-2** Principal component 1 (x-axis) and principal component 2 (y-axis) of the volatiles principal component analysis (PCA) color-coded by (a) land cover, (b) soil order, and categories representing the magnitude (low-high) of (c) total organic carbon (TOC), (d) elevation (e) distance from site to nearest coast, and (f) distance from site to nearest road
